# Evaluation of Whole-Genome Sequence Imputation Strategies in Korean Hanwoo Cattle

**DOI:** 10.3390/ani12172265

**Published:** 2022-09-01

**Authors:** Muhammad Yasir Nawaz, Priscila Arrigucci Bernardes, Rodrigo Pelicioni Savegnago, Dajeong Lim, Seung Hwan Lee, Cedric Gondro

**Affiliations:** 1 Genetics and Genome Sciences Graduate Program, Michigan State University, East Lansing, MI 48824, USA; 2 Department of Animal Science and Rural Development, Federal University of Santa Catarina, Florianopolis 88034-000, SC, Brazil; 3 Department of Animal Science, Michigan State University, East Lansing, MI 48824, USA; 4 Animal Genome & Bioinformatics Division, National Institute of Animal Science, RDA, Wanju 55365, Korea; 5 Division of Animal and Dairy Science, Chungnam National University, Daejeon 305764, Korea

**Keywords:** Hanwoo cattle, imputation accuracy, whole-genome sequence data

## Abstract

**Simple Summary:**

In this study, we evaluated various imputation strategies for the Korean Hanwoo cattle. We observed that a large reference panel consisting of many cattle breeds did not improve the imputation accuracy when compared to a proportionally small purebred Hanwoo reference. This was because the multi-breed reference did not contain animals sufficiently related to the Hanwoo to improve the accuracies and, although not detrimental, in effect, only added to the computational burden of the imputation. Despite the large multi-breed reference, when the Hanwoo were removed from the reference, the imputation accuracies were low. These results suggest additional sequencing efforts are needed for underrepresented breeds, particularly those less genetically related to the main European breeds.

**Abstract:**

This study evaluated the accuracy of sequence imputation in Hanwoo beef cattle using different reference panels: a large multi-breed reference with no Hanwoo (*n* = 6269), a much smaller Hanwoo purebred reference (*n* = 88), and both datasets combined (*n* = 6357). The target animals were 136 cattle both sequenced and genotyped with the Illumina BovineSNP50 v2 (50K). The average imputation accuracy measured by the Pearson correlation (R) was 0.695 with the multi-breed reference, 0.876 with the purebred Hanwoo, and 0.887 with the combined data; the average concordance rates (CR) were 88.16%, 94.49%, and 94.84%, respectively. The accuracy gains from adding a large multi-breed reference of 6269 samples to only 88 Hanwoo was marginal; however, the concordance rate for the heterozygotes decreased from 85% to 82%, and the concordance rate for fixed SNPs in Hanwoo also decreased from 99.98% to 98.73%. Although the multi-breed panel was large, it was not sufficiently representative of the breed for accurate imputation without the Hanwoo animals. Additionally, we evaluated the value of high-density 700K genotypes (*n* = 991) as an intermediary step in the imputation process. The imputation accuracy differences were negligible between a single-step imputation strategy from 50K directly to sequence and a two-step imputation approach (50K-700K-sequence). We also observed that imputed sequence data can be used as a reference panel for imputation (mean R = 0.9650, mean CR = 98.35%). Finally, we identified 31 poorly imputed genomic regions in the Hanwoo genome and demonstrated that imputation accuracies were particularly lower at the chromosomal ends.

## 1. Introduction

Whole-genome DNA sequence (WGS) data in livestock may lead to an increase in prediction accuracy, especially in less related populations [1,2], and a better resolution to identify causal loci for traits of interest [3]. Advances in next generation sequencing (NGS) technologies and a rapid decrease in sequencing costs have made WGS available for livestock species. Still, sequencing the large number of animals needed for genomic prediction is not yet economically feasible. The animal breeding industry and researchers are currently relying mostly on medium- and high-density SNP genotypes for association studies (GWAS) and for the genomic prediction of traits [4].

An alternative strategy to directly sequencing animals is through genotype imputation which is a cost-effective approach to acquire WGS data for a large number of animals. It is the process of inferring unknown genotypes (in silico) for animals genotyped at a lower density (e.g., 50K), using pedigree information and/or a set of reference animals genotyped at a higher density (e.g., 700K, WGS, etc.). Imputation can help improve genomic coverage, facilitate comparison and combination of studies that use different marker panels, increase the power to detect genetic associations by combining datasets from different studies, and guide fine-mapping of quantitative trait loci. Several studies have shown that imputed genotypes can lead to accurate genomic predictions and help identify quantitative trait loci (QTLs) [3,5,6].

Effective use of imputed genotypes in genomic selection requires that single-nucleotide polymorphisms (SNPs) are imputed with high accuracy [7]. Several factors affect imputation accuracy, for example, the number of animals in the reference population, SNP density in reference and target populations, minor allele frequency (MAF) of the SNPs to be imputed, the extent of linkage disequilibrium (LD), and the genetic structure of the population. Other factors that also affect the accuracy of imputation are the software used and its underlying imputation method, and somewhat more concerningly, even the metric used to evaluate imputation accuracy can lead to different interpretations [8]. To illustrate the latter, a study by Rowan et al. [9] observed that using a composite breed reference panel for Gelbvieh cattle resulted in higher accuracies for rare variants when measured by the quality score metric produced by the imputation software Minimac3, but no such gains were observed for the concordance rate and Pearson′s correlation between observed and imputed genotypes.

Previous research has shown that combining reference populations from different breeds to increase the size of the reference population may or may not be a good strategy to help increase the imputation accuracy. For example, a study showed that a reference population combining dairy and beef cattle breeds actually led to a decrease in the imputation accuracy from low-density SNPs to HD and suggested that it might be due to differences in the LD phase and haplotype dissimilarities between breeds [10]. However, in another study, a combined reference population of three closely related dairy breeds helped increase the sequence imputation accuracy when compared to a within breed reference [11]. Similarly, a large multi-breed reference population yielded higher imputation accuracies in Fleckvieh and Holstein target populations using the FImpute software, but there were no or negligible gains when using the Minimac software [12]. Another study reported modest gains in imputation accuracy for Gelbvieh cattle, which is a mixed ancestry breed, when using a large multi-breed reference [9]; however, the same study also observed a low imputation accuracy of individuals from breeds that were only sparsely represented and were distantly related to the reference population.

Imputation accuracies for cattle have been reported in several studies [13,14,15] but they are mostly limited to dairy breeds of European origin. To our knowledge there have not been any studies that evaluated the imputation strategies for less common breeds that are genetically distinct from the main European breeds and for which sequence information is rarely available to be used as a reference for imputation.

In this study, we report the accuracy of imputation from lower density genotypes (50K) to WGS in the Korean Hanwoo beef cattle, which are an East Asian taurine cattle breed more related to the Japanese Wagyu but very distinct from Western taurine cattle breeds. We also evaluated the imputation accuracies obtained when using single-breed (Hanwoo) and multi-breed reference panels to impute the Hanwoo genotypes. We further compared imputation accuracies obtained from one-step imputation (50K-WGS) and two-step imputation (50K-700K-WGS). Finally, we explored how imputation accuracy varies between common and rare SNP variants and across different genomic regions in the Hanwoo cattle.

## 2. Materials and Methods

### 2.1. Reference Genotype Data

The reference WGS data consisted of 201 Hanwoo cattle selected from key sites widely used in the national breeding program in Korea, the genomes from Run 9 of the 1000 Bull Genomes Project [16], plus other public sources of sequence consisting of 6292 samples from various breeds that included an additional 23 Hanwoo samples. The fastq files from the 201 Hanwoo were processed as described below and then combined with the 23 Hanwoo sequences from the 1000 Bull Genomes Project for a total of 224 Hanwoo samples in the Hanwoo reference.

**Figure 1:**
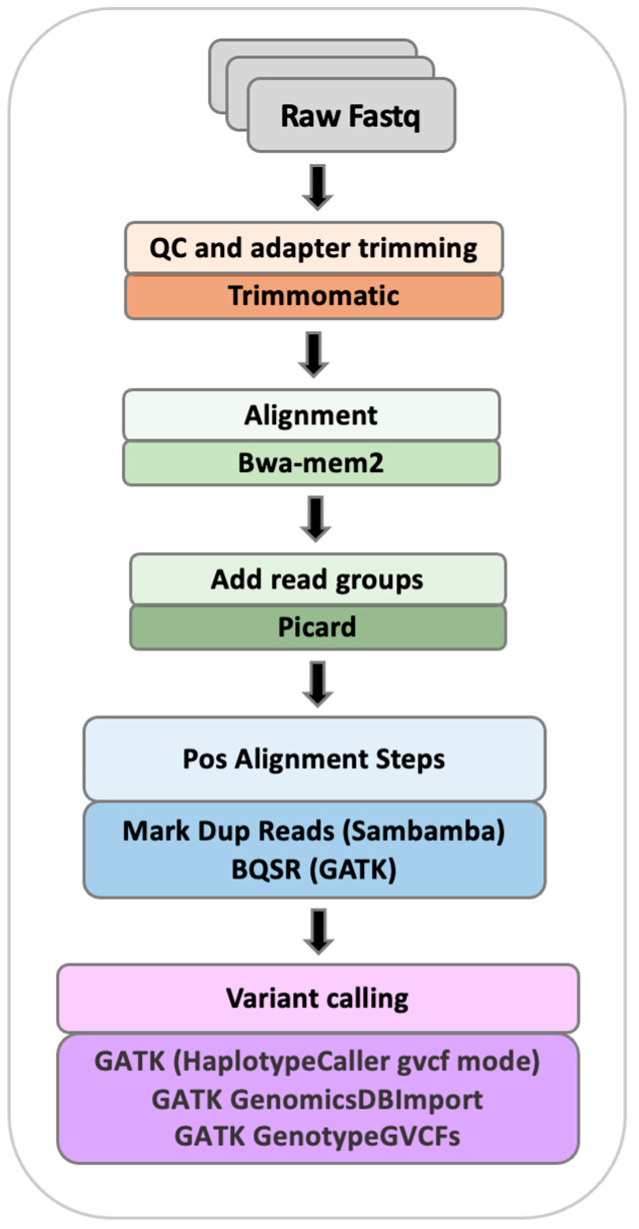
IVDP pipeline for variant calling from Illumina whole-genome short sequencing reads.

The variants in the reference panel were called from the Hanwoo sequencing short read files using IVDP—Integrated Variant Discovery Pipeline (https://github.com/rodrigopsav/IVDP accessed on 18 May 2022). IVDP follows the gold standard GATK pipeline for variant calling using whole-genome sequencing data (Figure 1) and is similar to the analysis pipeline used in the 1000 Bull Genomes Project. Standard read filtering and adapter removal were applied using trimmomatic [17]. The filtered reads were aligned onto the bovine ARS-UCD1.2 reference genome from Ensembl (GCA_002263795.2) with bwa-mem2 [18]. After that, duplicated reads were marked with sambamba markdup [19] and base quality score recalibration was carried out with GATK BaseRecalibratorSpark and ApplyBQSRSpark. Variant calling was performed using GATK (version 4.2.6.0) HaplotypeCaller with gVCF mode (HaplotypeCaller + GenomicsDBImport + GenotypeGVCFs commands). The variant calls were then filtered using the following criteria: must be biallelic across samples, must have variant and sample missingness ≤ 0.2, Phred-quality score (QUAL) ≥ 50; excluding single-nucleotide polymorphisms with QD < 2.0, MQ < 40.0, FS > 100.0, MQRankSum < −8.0, ReadPosRankSum < −20.0, ExcessHet > 54.69. No minor allele frequency filtering was performed at this stage as variants were called to align with variants in the 1000 Bulls run9 dataset [20].

**Figure 2:**
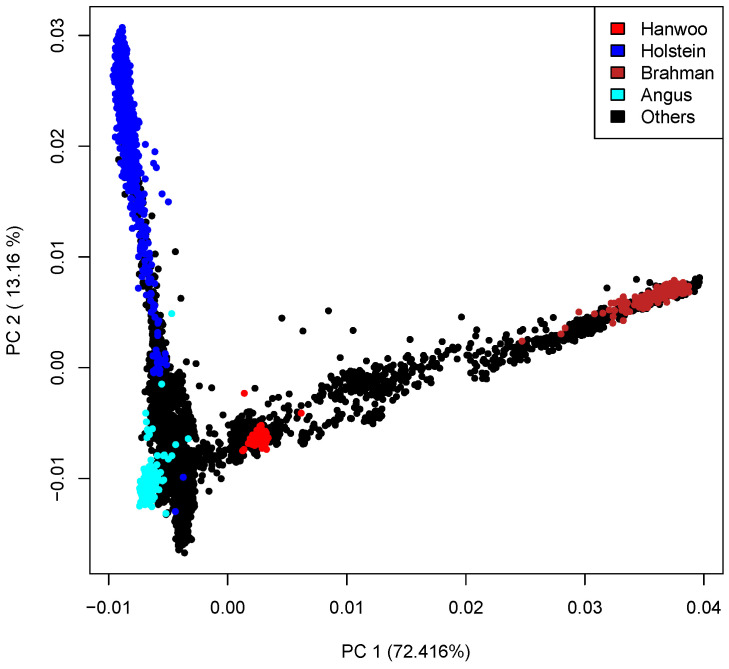
Plot of first two principal components of the large multi-breed reference including Hanwoo animals.

After filtering, the data were combined with the Hanwoo from the 1000 Bulls and the final reference panel ended up with 55,927,497 markers; however, 18,560,332 of these were monomorphic in the breed. For consistency, the same markers were used across all reference panel sets (a negligible number of new SNP variants detected only in the Hanwoo data were excluded). Finally, the reference panel was then phased with Beagle 5.4 software [20]. Only the 29 cattle autosomes were used in this study. Principal component analysis was performed to explore the genetic architecture of the multi-breed reference. The first principal component separated the taurine and indicine breeds while the second principal component separated the dairy and beef cattle breeds. Since the data consisted of more than 200 cattle breeds from around the world, only a few major breeds were highlighted in the figure (Figure 2).

### 2.2. Target Genotype Data

After sample quality control (samples with more than 10% of genotypes missing were excluded), a total of 9732 animals genotyped with the Illumina BovineSNP50 v2 (50K) and 991 animals genotyped with Illumina BovineHD chip (HD, 770K) were used as the target animals. Among these animals, there were 628 animals genotyped on both platforms and there were 136 animals that were both sequenced and genotyped on the 50K array. There was no overlap between the 700K and sequenced animals. The genotyped animals were from half-sib families and the average, minimum, and maximum numbers of progeny per sire was 9, 1, and 35, respectively. There were also 1783 animals in the target population that had at least one parent in the Hanwoo sequence reference population.

SNPs were realigned to the ARS reference assembly using a custom R script and the probe sequence information using blastn software (https://www.ncbi.nlm.nih.gov/books/NBK569856/, accessed on 18 May 2022). SNPs that could not be mapped to the reference, or if the REF/ALT alleles could not be unambiguously assigned, were excluded. SNP genotypes with missing rates greater than 20% or on non-autosomal chromosomes were also deleted. After SNP data filtering, 52,653 SNP from the 50K panel and 674,691 from the 700K were retained for imputation. From the 50K panel, there were 42,296 SNPs in common with the 700K and 46,734 SNPs in common with the sequence data. The 700K data had 668,998 SNPs in common with the sequence. Sporadic missing SNP genotypes (GC < 0.6) in the 50K and 700K datasets were then imputed and phased with Beagle 5.4 [21,22] using standard settings and the flags window = 300 and overlap = 100.

After initial comparison of one-step and two-step imputation strategies, the target animals for all other imputation approaches (Section 3.3 onwards) were only 136 animals whose sequence information was available for calculation of imputation accuracies. These 136 animals were previously phased to resolve haplotypes along with all other 50K animals, as described above.

### 2.3. Imputation

Imputation was carried out using Impute5 [23] with default parameters and one chromosome at a time. Two imputation strategies were evaluated: imputation of the 50K genotypes (*n* = 9732) directly to sequence using the Hanwoo purebred reference with 224 animals, and a two-step approach where the 50K genotypes were first imputed to the 700K panel with 991 animals and then imputed to the same reference sequence panel. For the two-step approach, after the 50K genotypes were imputed to 700K, and before imputing up to sequence, the genotypes were re-phased with Beagle, as recent work from Oget-Ebrad et al. [24] reported that Beagle is currently the best software to resolve haplotypes, which is critical for the accuracy of imputation.

We also explored how imputation accuracy varies in Hanwoo for within breed and multibreed reference populations. These analyses were performed only by one-step imputation as the imputation accuracy differences between the one- and two-step approaches were negligible (further details in the results and discussion). First, we set a baseline for imputation accuracy by using all available animals with WGS data as a reference to impute 136 animals genotyped at 50K level. This was a case of self-imputation as the target animals were also a part of reference dataset. In the next step, independent evaluation of different sequence reference panels was performed using: (1) only the purebred Hanwoo, (2) a large multi-breed reference without Hanwoo, and (3) both reference sets combined. Additionally, we increased the size of the purebred Hanwoo reference—the 136 Hanwoo with 50K and sequence data were randomly split into four groups of 34 target samples. For each of these groups, the target samples were excluded from the reference panel of 224 animals, leaving 190 for the reference which was then used for imputation. Imputed groups were then combined back into the 136 Hanwoo evaluation set and compared to the sequenced samples. Finally, we explored the possibility of using imputed sequence data as a reference for imputation. The imputed purebred Hanwoo data were used as a reference (*n* = 9596) after removing the target 136 animals from the imputed 50K dataset.

### 2.4. Evaluation of the Imputation

The 628 animals in common between the 50K and 700K datasets (674,691 SNPs) and the 136 animals in common between the 50K and sequence datasets (55,927,497 SNPs total but 18,560,332 monomorphic in Hanwoo) were used to calculate imputation accuracies. Accuracies were calculated as the correlation between the imputed and observed genotypes and as concordance rate (the percentage of correctly imputed genotypes). Accuracies per sample, per allele frequency, per genotype, and per SNP were also calculated. The pattern of imputation accuracy according to allele frequency was evaluated using average values of imputation accuracies in bins of 0.01. Differences between real and imputed datasets were estimated through a principal component analysis of the genomic relationship matrices (GRM).

## 3. Results and Discussion

### 3.1. Imputation from 50K to 700K in Hanwoo

The average imputation accuracy measured by the correlation between the imputed 50K and the reference HD was 0.996. At the animal level, correlations varied between 0.969 and 0.998; by SNP, correlations varied between −0.028 and 1 (2.2% of the SNPs had a correlation < 0.9). The minimum, average, and maximum concordance rates were 95.5%, 99.43%, and 99.76% by animal; by SNP, they were 26.43%, 99.43%, and 100%. Only 0.95% of the SNPs had a concordance rate below 90%. The correlation between real and imputed allele frequencies was 0.9996. At the genotype level, concordances were also very high, as shown in Table 1, and not surprisingly, the highest error rates were in the imputation results of the heterozygotes (~1% wrong). Concordances in allele frequency bins ranged between 0.9868 and 0.9995.

It is important to highlight that these imputation accuracies were overly optimistic and reflect the best-case scenario where the target samples were already part of the reference population. These results should not be construed as reflective of accuracies in usual circumstances where the target is not a subset of the reference. Our main objective at this point was primarily to ensure that the imputation from 50K to 700K was adequate for imputing up to sequence. A minor point of interest is that while most studies evaluate imputation by subsetting SNPs from a larger panel to a smaller one, here the same samples were independently genotyped on the two platforms. The discordance between observed genotypes was 0.115% and, of somewhat more concern, almost 10% of the missing genotypes in the 50K data, those that were imputed during the phasing step, differed from the genotypes in the 700K panel. The missing rate in the 628 animals was 8.37% and the overall discordance was 0.915%. In contrast, when the original 50K SNP panel is removed from the real and imputed 700K datasets, the discordance between them is lower at 0.542% which suggests a proportionally higher error rate of imputation of the missing genotypes during the phasing step with Beagle than of the imputation to higher density with Impute5. The haplotypes seem to have been well-resolved by the phasing step, which is crucial for the imputation, but again, some caution is warranted in not overinterpreting these accuracies since the target and reference sets had the same animals and it was expected that the haplotypes would align well between the two sets. Nevertheless, this analysis suggests the need for some attention when using Beagle to fill in missing genotypes.

### 3.2. Imputation from 50K to Sequence and Imputation from 50K to 700K to Sequence in Hanwoo

**Figure 3:**
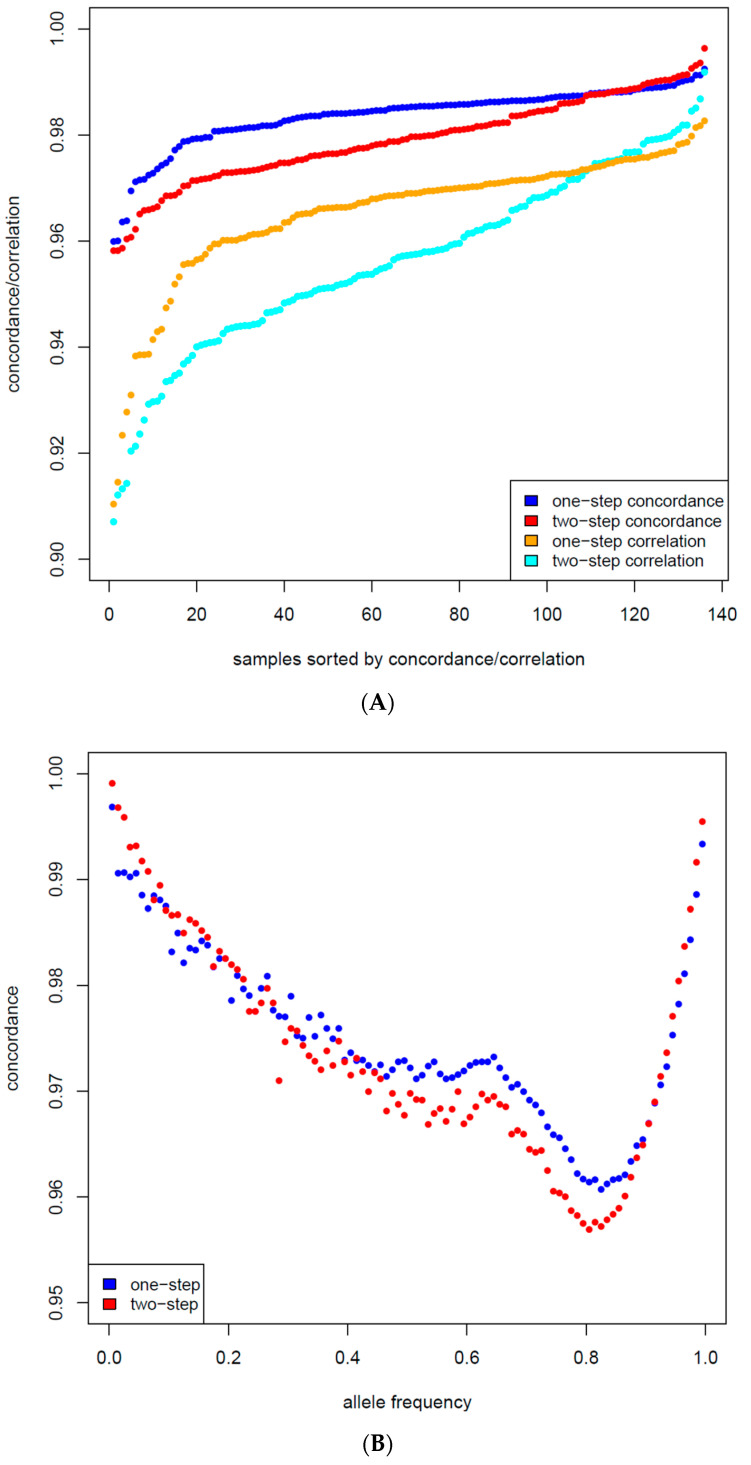
(**A**) Concordance and correlation of samples for one-step and two-step imputation. (**B**) Concordance by bins allele frequency (0.01 interval) for one-step and two-step imputation.

The average imputation accuracy measured by the correlation between the one-step imputed 50K and the reference sequence was 0.9653. At the animal level, correlations varied between 0.9104 and 0.9827 (Figure 3A); by SNP, correlations varied between −0.1 and 1 (18.74% of the SNPs had a correlation < 0.9, 8.8% < 0.8, and 4.5% < 0.7). The minimum, average, and maximum concordance rates were 95.99%, 98.36%, and 99.25% by animal (Figure 3A); by SNP, they were 0.0%, 98.36%, and 100%. 2.94% of the SNPs had a concordance rate below 90%. The correlation between real and imputed allele frequencies was 0.9968.

For the two-step imputation (50K-700K-WGS), the average imputation accuracy correlation was 0.9564. At the animal level, correlations varied between 0.9071 and 0.9918 (Figure 3A); by SNP, correlations varied between −0.1 and 1 (25.44% of the SNPs had a correlation < 0.9, 10.8% < 0.8, and 4.7% < 0.7). The minimum, average, and maximum concordance rates were 95.82%, 97.91%, and 99.63% by animal (Figure 3A); by SNP, they were 0.0%, 97.91%, and 100%. 3.45% of the SNPs had a concordance rate below 90%. The correlation between real and imputed allele frequencies was 0.9978.

At the genotype level, concordances were still high (Table 2) but noticeably lower than the 50K-700K imputation (Table 1). The error rates of the heterozygotes were 5.15% for the one-step imputation and 8.18% for the two-step. It is our view that the error rates of the heterozygotes are a preferable measure of imputation accuracy because they are the hardest to impute correctly. The concordances in allele frequency bins were similar for one-step and two-step imputation and ranged between 0.9607 and 0.9969, respectively (Figure 3B). We also observed that concordances were higher for variants at the two ends of allele frequency spectrum, i.e., for variants with low minor allele frequency (MAF), which is commonly observed in imputation studies [25].

When comparing one- and two-step imputation, 98.24% of the genotypes were identical. Out of the wrongly imputed genotypes, around half of these were the same between methods (~76 Million genotypes). Differences between the one- and two-step approaches were minimal, with a small advantage for the one-step approach. This goes against the consensus that it is preferable to first impute to a higher density panel and then up to sequence [26]. In other still unpublished work, we have noted that direct imputation certainly can yield high accuracies without first imputing to a high-density panel, provided that there is a large reference population and good coverage of the target haplotypes. In this particular instance, however, the higher accuracies from the direct imputation were more likely due to phasing errors of the 136 target animals when they were imputed from 50K up to 700K since they were not genotyped on the high-density array (in relation to the sequence haplotypes, the haplotype concordance of the 50K was slightly higher than the 700K at ~0.11%).

Keeping in mind that these were different animals and not the same sample sizes, the discordance between 50K genotypes and sequence was 0.445%, higher than between the 50K and 700K (0.115%). The discordance between the missing 50K genotypes was similar to what was observed in relation to the 700K at 6.85%.

**Figure 4:**
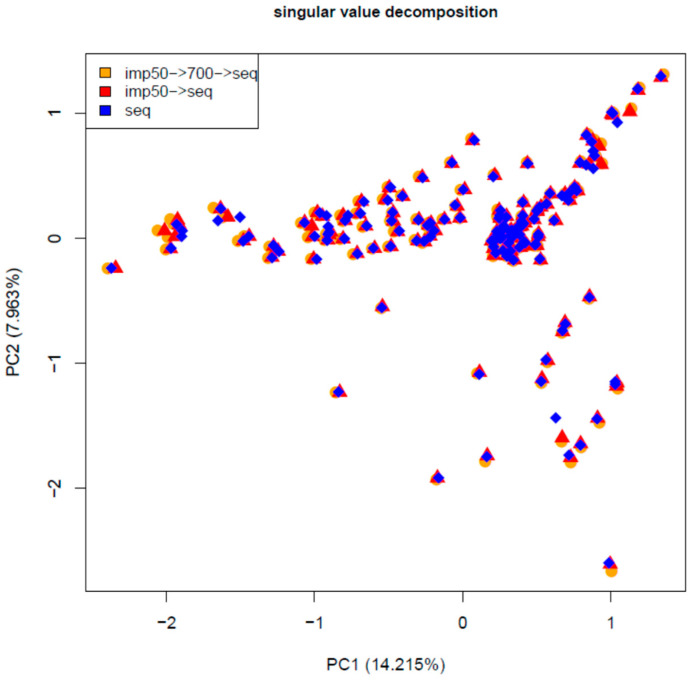
Plot of the first two principal components of the genomic relationship matrix of the sequence data and the imputed genotypes using the one- and two-step approaches.

The genomic relationship matrices of the sequence data and the two imputation approaches were very similar (Figure 4). The sums of squares of the deviation between the GRM of the sequence and the one-step was 0.3625 and for the two-step, it was slightly worse at 0.412. The effect of these deviations on the estimates of genomic breeding values should be negligible.

### 3.3. Evaluation of Imputation Accuracy with All Available Animals (Hanwoo and Multi-Breed)

To set a target for imputation accuracy and see how well the imputation pipeline can perform in a perfect world, the 224 Hanwoo animals plus other 6269 cattle genotypes from a broad range of breeds were used as the reference imputation panel. This reference panel is as good as it can be, as it contains the target 136 animals along with thousands of other sequenced animals. The accuracy results thus obtained will be used as a benchmark to achieve in subsequent imputations in this study. The average imputation accuracy measured by the correlation was 0.9711 (+0.0058 in relation to using only the Hanwoo samples as reference) and varied between 0.9058 and 0.9889 across samples. The average concordance rate was 98.62% (+0.26%, Table 3), varying between 95.78% and 99.50% across animals. An amount of 2.08% (−0.86%) of the SNPs had a concordance rate below 90%. The correlation between real and imputed allele frequencies was 0.9983 (+0.0015). The sums of squares of the deviation between the GRM of WGS and the imputed data were 0.3026.

The augmented reference set used in this section is almost 30 times larger than the Hanwoo-specific reference panel, but the imputation accuracy gains were only marginal. Of course, this is still a scenario where the target is a subset of the reference panel and the accuracies with the Hanwoo panel were already high anyway. It does, however, confirm that it is still beneficial, even if marginally, to increase the reference set by adding animals from other breeds and that the inclusion of very distantly related samples does not have a sizable negative effect on the accuracies; it also confirms that relatedness between target and reference is important for imputation accuracy, as discussed in more detail in the next section.

### 3.4. Evaluation of Imputation Accuracy with Independent Reference Sets

For a more realistic and independent evaluation of the imputation accuracy in routine settings, the 136 Hanwoo with WGS data were excluded from the reference panels. These animals were imputed directly up to WGS from their 50K original data. The accuracy with two reference panels was evaluated. One panel used the multi-breed genotypes without any Hanwoo (*n* = 6269); the other used the same panel plus the remaining 88 Hanwoo after exclusion of the 136 samples. The average imputation accuracy measured by the correlation was 0.6950 without the Hanwoo and 0.8873 with the Hanwoo. With reference sets in the same order, the correlations varied between 0.6670–0.9096 and 0.7973–0.9798 across samples. The average concordance rates were 88.16% and 94.84% varying between 86.42–95.89% and 91.55–99.03%, across animals. Amounts of 41.21% and 20.05% of the SNPs had a concordance rate below 90%. The correlation between real and imputed allele frequencies was 0.9322 and 0.9911. In total, 33.19% (18,560,332) of the 55,927,497 were fixed in the Hanwoo population. The concordance for the SNPs that should have been fixed in these individuals was 98.94% and 99.73%. Aside from the computational burden, the large number of SNPs that are not segregating in Hanwoo but are in other breeds did not introduce an excessive number of spuriously segregating genotypes within the breed; although, it should be noted that from the 18.5 million fixed SNPs, 31.48% and 16.60% had an imputed MAF > 0. The small effect on the concordance is due to the low frequencies of these incorrectly imputed SNPs (averages of 0.0118 and 0.0079). The bins concordances ranged between 0.6485–0.9794 and 0.8530–0.9936; the averages were 0.7259 and 0.8917.

At the genotype level, concordances were substantially lower, especially for heterozygotes (56.95%) when there were no Hanwoo in the reference. With Hanwoo included in the reference, concordances were much better but still around 82.15% for the heterozygotes (Table 4). The sums of squares of the deviation between the GRM of the sequence and the imputed data were 1.0552, and slightly lower (better) when Hanwoo were included in the reference at 0.9641.

Inclusion of Hanwoo samples in the imputation reference panel provided a substantial increase in the imputation accuracy, the gain in the correlation was 0.1923, and in the concordance was 6.68%. Even with a large reference population, when there were no samples of the targeted breed in the reference population, the imputation accuracies were rather low. Here, there were only 88 independent Hanwoo for the reference, but even this small number (1.38% of the total reference) already provided a substantial improvement in the accuracy. Hanwoo is an East Asian taurine breed quite distinct from other European breeds but more closely related to other Asian breeds, such as the Wagyu and Akaushi. The reference had a limited number of these breeds to assist with the imputation (e.g., only 174 Wagyu and a single Akaushi). There is still a need to increase the representation of less European-centric breeds in sequencing efforts to improve the imputation results of these breeds.

To better separate the contribution of within and between breed sequence data to the accuracy of imputation, we also used only the 88 Hanwoo as a reference. The average imputation accuracy measured by the correlation was 0.8759, varying between 0.8205 and 0.9421 across samples. The average imputation accuracy measured by the concordance was 94.49%, varying between 92.60% and 97.43%. Similar to the results discussed in the previous section, the accuracy gains from adding all the other 6269 samples were marginal at +0.0114 for the correlation and +0.35% for the concordance. The correlation between real and imputed allele frequencies was 0.9807 (−0.0105), but the concordance of the fixed SNPs correctly imputed increased to 99.98% without the noise introduced by the variants segregating in the other breeds. An amount of 21.91% (1.86% worse) of the SNPs had a concordance rate below 90%. The allele frequency bins concordances ranged between 0.8717–0.9653; the average was 0.8922. The genotype concordances (Table 5) were similar to those obtained with the multi-breed reference including Hanwoo, even slightly better as the heterozygote error rates were lower at 14.18% (but ~1% loss of concordance for each of the homozygotes).

### 3.5. Evaluation of Imputation Accuracy with a Larger and Independent Hanwoo Only Reference Set

Since there was a neglegible overall improvement in the imputation accuracy using the multi-breed reference, we opted to use only the purebred Hanwoo data (*n* = 190 in four independent chunks) as the final reference panel for this study. The average imputation accuracy measured by the correlation was 0.9336 and varied between 0.8459 and 0.9741 across samples. The average concordance rate was 96.88%, varying between 93.50% and 98.78% across animals. An amount of 8.35% of the SNPs had a concordance rate below 90%. The correlation between real and imputed allele frequencies was 0.9960. The sums of squares of the deviation between the GRM of the sequence and the imputed data were 0.4923. At the genotype level, concordances were high (Table 6) and the error rates of the heterozygotes were 10.47%. The allele frequency bins concordances ranged between 0.9178 and 0.9983; the average was 0.9431. Overall, this reference set seems adequate for accurate sequence imputation in Hanwoo, apart from some genomic regions of lower accuracy, which are discussed below (Section 3.7).

### 3.6. Evaluation of Imputation Accuracy with an Imputed Hanwoo Reference Set

The real imputation accuracy in the samples without sequence data is unknown. As an empirical evaluation, we used the imputed sequence data of the 50K samples (one-step imputation) as a reference panel (*n* = 9596) to impute the 136 animals with sequence data. Target samples were removed from the imputed sequence data and their original 50K genotypes were used as the target, after phasing with Beagle using the other phased 50K genotypes (the phasing reference panel). There is, of course, still some circularity to this argument as the 136 target samples accounted for 61% of the initial reference dataset used to impute the 50K genotypes at the first place, i.e., even though none of the samples in the reference overlaps with the target, the latter was initially used to impute them—this should lead to some upwards bias in the imputation accuracies. The average imputation accuracy measured by the correlation was 0.9650 and varied between 0.9103 and 0.9829 across samples. The average concordance rate was 98.35%, varying between 95.98% and 99.25% across animals, and only 2.99% of the SNPs had a concordance rate below 90%. The correlation between real and imputed allele frequencies was 0.9967. The sums of squares of the deviation between the GRM of the sequence and the imputed data were only 0.3786. At the genotype level, concordances were high, and the error rates of the heterozygotes were only 5.21% (Table 7). The frequency bins concordances ranged between 0.9605 and 0.9967.

This was effectively the best imputation accuracy, almost on par with having the animals in the reference set itself along with a large multi-breed reference set (Table 3 and Table S1). Again, accounting for the lack of independence in how the imputed reference samples were originally imputed, this does suggest the possibility of using imputed data along with real data to improve imputation accuracies. How well this would work in practice will be a function of the value of a larger number of haplotypes in the reference, offset by the errors in the imputed data—but that is a topic for future work.

### 3.7. Evaluation of Imputation Accuracy across Chromosomal Regions

**Figure 5:**
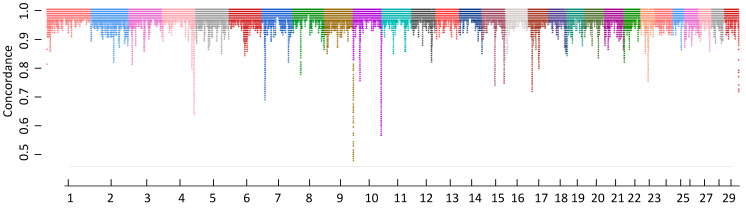
Smoothed concordance rate across 29 autosomes in Hanwoo cattle.

**Figure 6:**
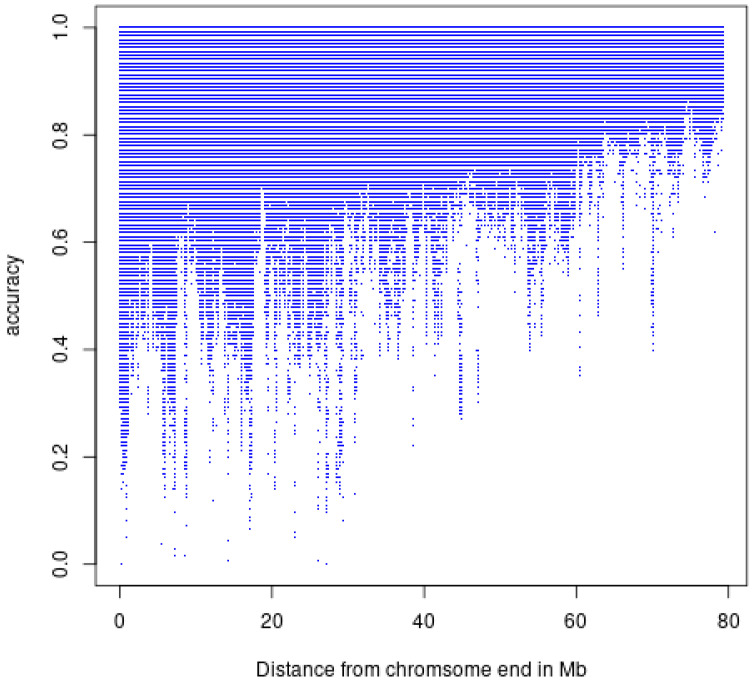
Plot of imputation accuracy against distance of SNPs from nearest end of chromosome (Mb).

We computed the running median of imputation accuracies in windows of 1001 SNPs to detect poorly imputed chromosomal regions across the Hanwoo genome. SNPs falling in the 0.1 percentile (CR cutoff value 0.853, *n* = 48,438) were used to identify genomic regions with poor concordance rates that contained two or more SNPs. CHR 4 contained the highest number of poorly imputed SNPs (*n* = 15,339) followed by CHR 17 (*n* = 9177), 10 (5326), and 7 (*n* = 4747). Poorly imputed SNPs located more than 1 Mb apart were considered as a separate genomic region. The five longest regions were located on CHR 17, 10, 15, 23, and 4. The longest one was on CHR 17 and spanned the region between 37,810,468 and 39,363,461 BP. Although imputation accuracies were generally high, there were some noticeable regions with poor imputation accuracies especially close to the ends of chromosomes due to higher recombination rates [27] (Figure 5 and Figure 6).

Additionally, highly polymorphic genomic regions, such as the major histocompatibility complex (MHC), are intrinsically difficult to impute due to the high number of repetitive elements, a greater diversity of haplotypes, and a complex LD structure. For example, in this study we did identify one such region on CHR 23 that overlaps with the bovine leukocyte antigen (BoLA) class II that encodes genes that perform similar functions across species but are structurally different. Other poorly imputed genomic regions and the number of SNPs in those regions are presented in Table 8. Such intrinsically difficult-to-impute genomic regions have also been reported in Flekvieh, Holstein, and Nelore cattle [21,22,23] and should be taken into account in studies that use impute sequence data. Additional reasons for some regions having low imputation accuracy can be due to a low coverage by SNP panels and a high sequence variant density in a region, high GC content, and assembly errors.

Previous studies have suggested the use of a multi-breed reference population for whole-genome imputation, but the target populations in those studies consisted of either admixed populations or cattle breeds closely related to the reference breeds [9,11,12]. Our results collectively indicate that the imputation accuracy in Hanwoo was largely driven by within breed imputation. The large multi-breed reference panel added very little to the imputation accuracy beyond what was obtained by simply using the closely related samples from the breed itself. This was mainly because the 1000 Bulls sequence data mostly contained cattle breeds of European origin, while haplotype diversity and genetic architecture of breeds originating from Asia and Africa were poorly represented. On the upside, however, there was no clear indication of the diverse reference having a negative effect on the overall imputation accuracy, although it did increase the imputation error rates of heterozygotes and falsely detected some rare variants that did not actually exist in the target population. There is some evidence in the literature that adding a small number of animals from various breeds to a small reference panel can adversely affect accuracy. However, a large number of multi-breed animals in the reference would help increase accuracy [28]. Therefore, the ratio of within breed to multi-breed animals in the reference is also important. The large and diverse multi-breed reference in our study also added considerably to computational burden considering the fact that it was ~40 times greater than the within breed reference. These findings suggest that in the absence of an adequately sized breed-specific panel, a large multi-breed reference can be used, but the error rates will be high. This should not overly affect genomic prediction (although there is limited value in using sequence data to estimate GEBVs anyway [1,26,29]), but could be more consequential for association studies.

One aspect we have not yet considered is the Impute5 imputation info scores. These are estimates of the ratio between the observed and expected statistical information [30]. This measure aims to serve as a guide as to the reliability of the imputed genotypes, but it is quite dependent on the estimated allele frequency of the imputed genotypes. Hard filters, e.g., R < 0.6, are commonly used but it does not seem to align well with observed concordance values. For example, in our dataset, the correlation between the info score and SNP concordance is only 0.2598. The correlation with allele frequencies is even lower at 0.0908. If the 0.6 filter cutoff was used on these data, the average concordance for the filtered-out data would be 0.9574 and for the selected data, it would be 0.9715 (and would remove ~20% of the genotypes). The percentage of SNPs with a concordance below 0.9 would only improve from 8.35% to 7.20%. Within allele frequency bins (in 0.01 intervals), the minimum concordance was 0.9311 and the highest 0.9983; however, the correlation of these concordance means in the bins and allele frequencies is also low at 0.0603 and not associated with allele frequencies. The metric does seem more accurate with more stringent cutoffs though; for example, on the extreme, if the data are filtered for an R = 1, then the concordance mean in the subset is 99.99%, and only 0.015% of the SNPs will have a concordance < 0.9, but only 30.38% of the genotypes will be kept and, much more problematically, only a fraction of these will have an MAF > 0.01. In this work, we could not find a meaningful way to use the info scores that would help to filter out SNPs with low imputation accuracy.

## 4. Conclusions

We have demonstrated that imputation with a small reference population closely related to the target population is more accurate than a large multi-breed reference with distantly related animals. Without any representation of the target breed in the reference population, the imputation accuracies for Hanwoo were substantially lower, which is due to the high genetic distances between the Korean breed and other breeds. The addition of a relatively small number of Hanwoo animals to the large multi-breed reference panel considerably improved the imputation accuracy. However, the accuracies of the rare variants were still slightly lower than those obtained when using only a purebred Hanwoo reference. It implies that even though a large variety of dairy and beef breeds from around the world have already been sequenced, there is still a need to increase the representation of less European-centric breeds in sequencing efforts to improve the imputation of these breeds. Furthermore, while a two-step imputation strategy is usually the suggested approach, in this work, the differences in imputation accuracy when compared to single-step imputation were negligible. We also observed that imputed genotypes can be used as the reference panel which suggests that, for example, HD genotypes could be imputed to sequence level and then, included along with the original sequence reference panel for use in one step imputation approach. This could reduce the computational burden of the two-step approach and potentially improve imputation accuracies when the number of informative animals in the WGS reference panel is small. Lastly, we also identified poorly imputed genomic regions in Hanwoo cattle that should be accounted for when the imputed data are used in other projects—particularly in genome-wide association studies.

## Figures and Tables

**Table 1:** Concordance rate in percentage between imputed and real genotypes for imputation from 50K to 700K (991 700K reference).

	AA_imp_	AB_imp_	BB_imp_
**AA_ref_**	99.57	0.41	0.02
**AB_ref_**	0.48	99.03	0.48
**BB_ref_**	0.03	0.33	99.65

_imp:_ imputed genotypes; _ref:_ reference genotypes.

**Table 2:** Concordance rate in percentage between imputed sequence and real sequence of 136 animals for one-step (224 WGS reference) and two-step imputation (224 WGS and 991 700K reference).

One-Step				Two-Step			
	AA_imp_	AB_imp_	BB_imp_		AA_imp_	AB_imp_	BB_imp_
**AA_ref_**	97.14	2.65	0.22	**AA_ref_**	95.69	3.98	0.33
**AB_ref_**	1.10	94.85	4.05	**AB_ref_**	1.4	91.82	6.78
**BB_ref_**	0.03	1.07	98.91	**BB_ref_**	0.02	1.09	98.89

_imp:_ imputed genotypes; _ref:_ reference genotypes.

**Table 3:** Concordance rate in percentage between imputed and real genotypes for 224 Hanwoo and 6269 multi-breed animals in reference.

	AA_imp_	AB_imp_	BB_imp_
**AA_ref_**	97.75	2.06	0.19
**AB_ref_**	0.88	94.8	4.33
**BB_ref_**	0.01	0.78	99.2

_imp_ imputed genotypes; _ref_ reference genotypes.

**Table 4:** Concordance rate in percentage between imputed and real genotypes for multi-breed reference panels with (88 Hanwoo, 6269 multi-breed) and without Hanwoo (6269 multi-breed) samples.

Multi-Breed without Hanwoo				Multi-Breed with Hanwoo			
	AA_imp_	AB_imp_	BB_imp_		AA_imp_	AB_imp_	BB_imp_
**AA_ref_**	76.03	18.97	5.01	**AA_ref_**	90.45	8.84	0.71
**AB_ref_**	12.29	56.95	30.76	**AB_ref_**	5.15	82.15	12.7
**BB_ref_**	0.71	8.09	91.2	**BB_ref_**	0.1	3.23	96.67

_imp:_ imputed genotypes; _ref:_ reference genotypes.

**Table 5:** Concordance rate in percentage between imputed and real genotypes with a reference panel of 88 purebred Hanwoo.

	AA_imp_	AB_imp_	BB_imp_
**AA_ref_**	89.21	9.93	0.86
**AB_ref_**	4.63	85.82	9.54
**BB_ref_**	0.18	4.14	95.68

_imp:_ imputed genotypes; _ref:_ reference genotypes.

**Table 6:** Concordance rate in percentage between imputed and real genotypes with a reference panel of 190 purebred Hanwoo.

	AA_imp_	AB_imp_	BB_imp_
**AA_ref_**	93.69	5.91	0.40
**AB_ref_**	2.47	89.54	8.00
**BB_ref_**	0.04	1.93	98.03

_imp:_ imputed genotypes; _ref:_ reference genotypes.

**Table 7:** Concordance rate in percentage between imputed and real genotypes of 136 target animals with a reference panel of 9596 purebred Hanwoo that were previously imputed from 50K to sequence.

	AA_imp_	AB_imp_	BB_imp_
**AA_ref_**	97.11	2.67	0.22
**AB_ref_**	1.11	94.79	4.10
**BB_ref_**	0.03	1.07	98.90

_imp:_ imputed genotypes; _ref:_ reference genotypes.

**Table 8:** Chromosomal position of poorly imputed genomic regions in Hanwoo.

CHR	Start (BP)	End (BP)	Length (BP)	No. of SNPs
2	81,496,310	81,511,148	14,838	273
3	11,716,513	11,776,690	60,177	452
4	105,573,863	105,589,511	15,648	1422
4	112,830,880	113,341,416	510,536	13,917
5	92,617,024	92,629,684	12,660	236
6	55,737,025	55,758,674	21,649	508
7	10,758,277	11,039,089	280,812	4584
7	95,736,166	95,742,201	6035	163
8	29,160,922	29,183,317	22,395	559
9	10,593,065	10,596,144	3079	59
9	104,358,861	104,380,205	21,344	671
10	459,493	521,352	61,859	846
10	22,908,467	23,406,397	497,930	2334
10	100,209,890	101,216,542	1,006,652	2146
11	40,903,028	40,905,284	2256	72
12	70,443,588	70,455,007	11,419	713
12	71,728,153	71,735,710	7557	719
14	82,178,440	82,180,992	2552	60
15	45,854,158	46,106,343	252,185	3506
15	78,379,540	79,226,472	846,932	398
16	372,932	418,633	45,701	1473
16	5,775,482	5,778,367	2885	53
17	14,049,911	14,159,496	109,585	4297
17	37,810,468	39,363,461	1,552,993	4880
18	62,688,950	62,690,650	1700	5
19	927,489	945,870	18,381	537
20	49,746,665	49,760,276	13,611	432
22	897,334	922,574	25,240	738
23	15,964,918	15,976,090	11,172	138
23	25,875,394	26,511,965	636,571	2223
29	51,092,637	51,093,495	858	23

## Data Availability

The data that support the findings of this study were available from the Rural Development Administration, Republic of Korea. Restrictions apply to the availability of these data, which were used under license for this study.

## Supplementary Materials

The following supporting information can be downloaded at: https://www.mdpi.com/article/10.3390/ani12172265/s1; Table S1: Summary statistics of imputation accuracy for all imputation scenarios.
